# Effect of Intra CA1 and Intraperitoneal Administration of Opioid Receptor Modulating Agents on The Anxiolytic Properties of Nano and Conventional ZnO in Male Rats

**Published:** 2014-05-25

**Authors:** Mozhgan Torabi, Mahnaz Kesmati, Hooman Eshagh Harooni, Hosein Najafzadeh Varzi

**Affiliations:** 1 Department of Biology, Faculty of Sciences, Shahid Chamran University, Ahvaz, Iran; 2 Department of Pharmacology, Faculty of Veterinary Medicine, Shahid Chamran University, Ahvaz, Iran

**Keywords:** Nanoparticls, Zinc Oxide, Anxiety, Opioid, Hippocampus

## Abstract

**Objective:**

Nano components are today’s new wonder material. However, the safety or toxicity of these components in humans is not yet clear. In a previous study we indicated that nano ZnO (nZnO) has a stronger anxiolytic effect compared to the conventional ZnO (cZnO). The present study was designed to evaluate the intraperitoneal administration of an opioidergic receptor agonist and antagonist of as well as the intra CA1 administration of an opioidergic receptor antagonist on the anxiolytic properties of nano and conventional ZnO in adult male Wistar rats.

**Materials and Methods:**

In this experimental study, rats received drugs via two modes of injection; intraperitoneal (IP.) and intra CA1 (intra hippocampus, CA1 area). Firstly, nZnO (5, 10, 20 mg/kg), cZnO (5, 10, 20 mg/kg), morphine 6 mg/kg, and naloxone 1 mg/kg were injected IP and naloxone 1µg/rat was injected intra CA1. Subsequently, morphine and na- loxone (IP and intra CA1) were co-injected with the effective dose of nZnO and cZnO. An elevated plus maze was used to evaluate anxiety related behavior and anxiety parameters 30 minutes after the second injection.

**Results:**

The results indicated that the anxiolytic effects of nZnO 5 mg/kg and cZnO 10 mg/kg were equal. When injected intraperitoneally, naloxone increased anxiety but did not inhibit the anxiolytic effect of nZnO and cZnO. The anxiolytic effects of morphine potentiated the anxio- lytic effects of ZnO, particularly nZno. When introduced via intra CA1 injection naloxone alone had no effect on anxiety behaviors and did not inhibit the anxiolytic effect of nZnO.

**Conclusion:**

It seems that the opioidergic system activity involved in the anxiolytic effect of nano and conventional ZnO may operate through shared and unshared pathways.

## Introduction

Zinc is an element essential for the correct functioning of the brain and other body organs (1). In the peripheral and central nervous system Zinc modulates many receptors (2). It is concentrated mainly in the hippocampus; in the subiculum of the dentate gyrus and in the accessory olfactory bulb (3).

Approximately half of the world’s population does not get adequate zinc (4, 5). Some studies have shown that zinc deficiency might induce anxiety-like behavior in animals (6). It has been indicated that dietary zinc deficiency in laboratory animals could cause anxiety (7), while feeding with organic and inorganic zinc supplements, such as zinc sulphate, conventional ZnO (cZnO) and zinc-methionine can be effective in reducing this anxiety (8).

Anxiety disorder is a common mental health

The recent development and expansion of nanotechnology has resulted in a rapid increase in the use of nanoparticles to replace normal scale particles (12). Due to its unique properties, nano ZnO (nZnO) is one of the most widely used of the engineered metal oxide nano materials (13). nZnO has attracted the attention of many researchers in medicine and pharmacology because of its potential therapeutic applications, for example as a drug delivery agent or as an anticancer drug, and its potential in imaging (14-16).

Many studies have reported that opioidergic system activity could influence anxiety related behavior and (17, 18) in turn be influenced by zinc homeostasis in body (19, 20). Intrathecal injection of zinc has been shown to inhibit the development of acute morphine tolerance (19). Zinc concentration is lower in the cerebrospinal fluid (CSF) of ex-heroin addicts and contributes to a long term state of dependence in these individuals (21). In our previous study we indicated that the anxiolytic effect of nZnO is much higher than its conventional form (22). In the present study intra CA1 (the hippocampus being one of the main zinc storage regions) and peripheral injections of drugs that modulate the effect of the opioidergic system on the anxiolytic properties of nano and conventional ZnO were investigated.

## Materials and Methods

### Animal care

In this experimental study, the subjects were male albino Wistar rats weighing 220 ± 20 g purchased from the animal house of the Medical Science Department of the Joundi Shapor University of Ahvaz, Iran. Rats were accommodated for more than a week in a room at 24 ± 1˚C, with controlled 12/12 hours light-dark cycles. They were housed in polypropylene cages (4 per cage). Food and drinking water were freely available except during the brief test periods. In each experiment 6-8 animals were used. Each animal was used once only and experiments undertaken during the light phase. All procedures were carried out in accordance with the institutional guidelines for animal care and use at the Shahid Chamran University of Ahvaz (23).

### Drugs

**Fig 1:**
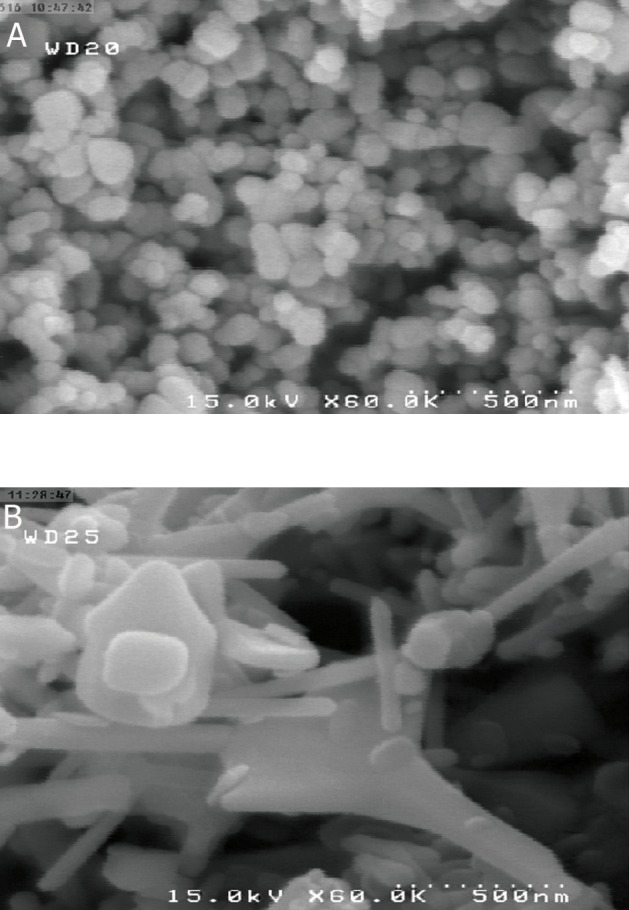
Scanning electron microscopy image of A(dry powder of nZno) and B(dry powder of cZnO). Images show difference between the size of nano and conventional form of ZnO powder.

The drugs used in the study were nZnO (Lolitech Co, Germany), cZnO (Merc Co, Germany), morphine sulphate (Temad Co, Iran) and naloxone hydrochloride (Sigma Co, Germany). nZnO was prepared by sonication for 15 minutes in an ultrasonic bath. The resulting suspension was shaken for 1 minute before each injection. Morphine sulphate and naloxone hydrochloride were dissolved in 0.9% saline. For peripheral administration, all drugs were injected intraperitoneally at concentrations measured as mg/kg in volumes of 1ml/kg. The control group received 1ml/kg 0.9% saline. For central administration naloxone 1 μg/rat or saline 1μl/rat was injected into the intra CA1 of the dorsal hippocampus. The interval time between central and peripheral injections was 5 minutes and between two peripheral injections was 15 minutes. Figure 1 shows scanning electron microscopy images of nZnO and cZnO powder for determination of the size of these particles.

### Animal surgery

Animals were anesthetized by intraperitoneal administration of ketamine hydrochloride (60 mg/ kg) and xylezine (4 mg/kg) and were subsequently placed in a stereotaxic apparatus. A stainless steel cannula (22 gages) was implanted in the dorsal hippocampus. Coordinates for cannula implantation in the CA1 of the dorsal hippocampus were anterocaudal: −2.6 mm; lateral: ± 2 (with respect to the bregma), vertical: 3.3 mm (from the dura) according to the atlas of Paxinos and Watson (24). The cannulas were anchored to the skull with two jeweler’s screws and acrylic dental cement. After surgery, the rats were allowed to recover for 7 days. The drug solutions were injected over a period of 1 minute through an internal cannula (27 gage) connected by polyethylene tubing to a 2 μl Hamilton syringe. The injection cannula was left in place for an additional 1 minute before being slowly withdrawn. The left and right hippocampi were injected with 0.5 μl of solution on each side (1 μl/rat) over a 1 minute period.

### Elevated plus maze

All behavioural testing took place in a dimly lit room. Animals adapted to the testing room over a 1 hour period prior to testing. The wooden plus maze Shahid Chamran University of Ahvaz, Iran) consisted of two open arms (50×10 cm), and two closed arms of the same size but with 40 cm high end and side walls. The arms were connected by a central 10×10 cm area and there were no walls on the open arms. The height of the elevated plus maze (EPM) above the floor was 50 cm. Rats were placed in the centre of the EPM with their head facing an open arm and left undisturbed for 5 minutes. Rats were then removed and returned to their home cages. The experimental sessions were recorded by camera and analyzed later (by maze router software Co, Iran). A rat was considered to be on the central platform when at least two paws were on it and on an arm whenever all four paws were on it. Percent of time spent in open arms [open arm time OAT%: (time in open arm/time in open + closed arm) ×100] and percent of open arm entries [open arm entries OAE%: (number of open arm entries/ number of open + closed arm entries) ×100] were used as a measure of anxiety. The distance travelled in the closed and open arms in 5 minutes was used as a measure of locomotor activity by maze router soft ware. In all experiments the interval time between injections and tests was 30 minutes (25).

### Statistical analysis

Data were expressed as mean ± SEM. Student’s t test was used for comparison of the means of unpaired data. ANOVA was used for multiple comparisons between groups and Student-Newman- Keuls post hoc test was performed using Instate 3 software. Differences with a p value of <0.05 between experimental groups at each point were considered statistically significant.

## Results

### Comparison between the anxiolytic effects of nano and conventional ZnO (5, 10, 20 mg/kg)

**Fig 2:**
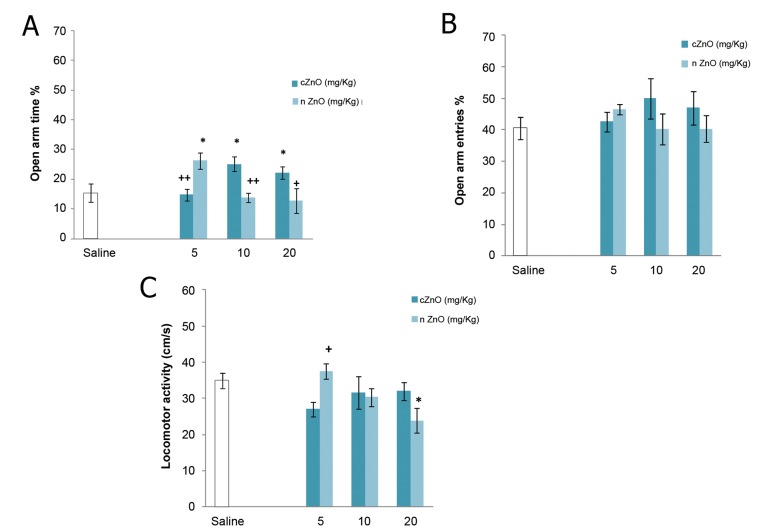
Comparison between anxiolytic effects of nano and conventional ZnO (5, 10, 20 mg/kg). Each bar shows mean ± SEM, *; p<0.05 in the treatment group compared to the control group, +; p<0.05, ++; p<0.01 for comparison between equal doses.

Figure 2 shows that cZnO 10 and 20 mg/kg and nZnO 5 mg/kg significantly increased OAT% (p<0.05), indicating that these doses have an anxiolytic effect. Significant differences in OAT% were also observed when equal doses (5, 10 mg/kg p<0.01 and 20 mg/kg p<0.05) of nZnO and cZnO were compared. There were no significant differences in OAE% between nZnO or cZnO groups and controls.

NZnO 20 mg/kg significantly reduced locomotor activity in treated rats compared to controls (p<0.05) and the difference between 5 mg/kg nZnO and 5 mg/ kg cZnO was significant (p<0.05). As these results indicate nZnO 5mg/kg and cZnO 10 mg/kg have the greatest anxiolytic effect, we selected them for the following experiments.

### The effect of morphine sulphate 6 m/kg and/or naloxone hydrochloride 1 mg/kg alone and coinjected with nZnO 5 mg/kg and/or cZnO 10 mg/ kg on anxiety related behaviors

Morphine exhibited an anxiolytic effect while naloxone exhibited an anxiogenic effect which significantly increased and decreased OAT% respectively in treated rats compared to controls (p<0.01, p<0.05). Morphine also increased OAE% in treated rats compared to controls (p<0.05). Both drugs had no effect on locomotor activity.

**Fig 3:**
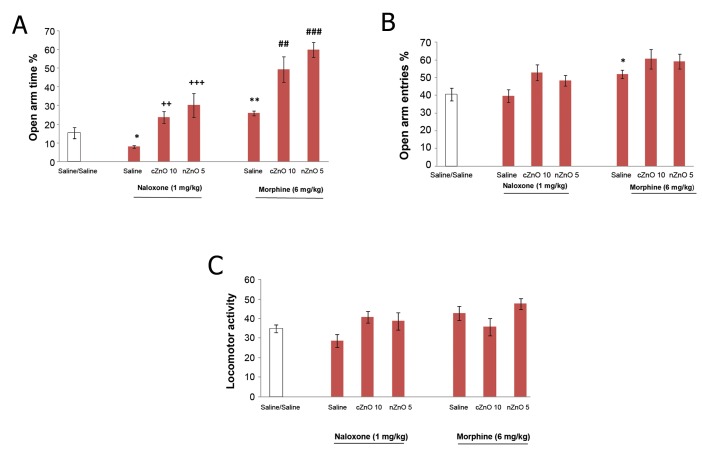
The effect of morphine sulphate 6 m/kg and/or naloxone hydrochloride 1 mg/kg alone and co-injected with nZnO 5 mg/kg and/or cZnO 10 mg/kg on anxiety related behaviors. Each bar is mean ± SEM. *; p<0.05, **; p<0.01 for the treatment group compared to the saline/saline control group, ++; p<0.05, +++; p<0.01 compared to the naloxone/ saline control group, and ##; p<0.01, ###; p<0.001 compared to morphine/saline control group.

The anxiolytic effect of nZnO and cZnO was not affected by the presence of naloxone. As shown in figure 3, nZnO and cZnO in the presence of naloxone significantly increased OAT% in treated rats compared to the naloxone/ saline group (p<0.001, p<0.01).

NZnO and cZnO significantly increased OAT% (p<0.001, p<0.01 respectively) in treated rats compared to the morphine/saline group, but had no effect on locomotor activity. These results indicated that nZnO (5 mg/kg) and cZnO (10 mg/kg) could increase the anxiolytic effects of morphine (Fig 3).

### The anxiolytic effect of nZnO (5 mg/kg) and cZnO (10 mg/kg) in the presence of intra CA1 administration of naloxone

**Fig 4:**
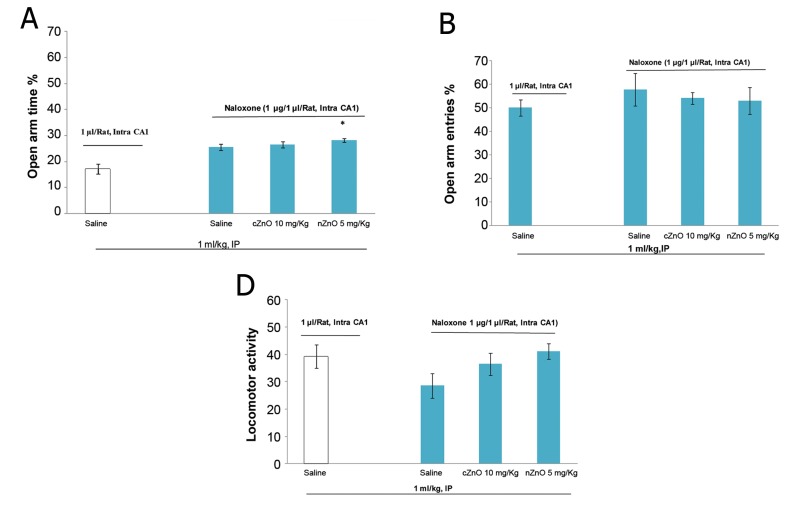
Effect of nZnO (5 mg/kg) and cZnO (10 mg/kg) in presence of naloxone (1 μg/Rat) on anxiety-related behaviors and locomotor activity. *; P<0.05 treatment group in comparison with saline control group. Each bar is mean ± SEM.

The intra CA1 administration of naloxone alone did not affect the anxiety parameters and did not inhibit the anxiolytic properties of nZnO (5 mg/kg). Conventional ZnO had no effect on the anxiety parameters either when injected alone or when co-injected with the intra CA1 administration of naloxone (Fig 4).

## Discussion

The results of our study show that nZnO 5 mg/ kg and cZnO 10 and 20 mg/kg reduced anxiety related behaviors without any change in locomotor activity. These results support those of previous studies which have shown that high levels of zinc supplements, such as zinc-methionine, ZnSO_4_ and ZnO reduced anxiety in rats during the elevated plus maze test (8). It has also been shown that dietary zinc deficiency in mice induced anxietyrelated behavior in the novelty suppressed feeding test (7).

Figure 2 further demonstrates that equal doses of nZnO and cZnO have different effects on anxiety behaviors and that the effective dose for nZnO is half that for cZnO. These effects may be due to the small size of nZnO and different physicochemical properties compared with the conventional form. The main characteristic of nano materials is their small size (26). This can modify the physicochemical properties of the material as well as create the opportunity for increased uptake and interaction with biological tissues (27, 28). Due to their small size nano particles of ZnO have both greater mobility and uptake across biological membranes (26, 28). The increase in surface area increases the number of reactive groups on the particle surface and makes this form more reactive than the conventional form (29, 30).

Electrophysiological studies have shown that zinc is an antagonist for the N-methyl-D-aspartate receptor (NMDA) glutamate receptor and weakens this receptor’s mediated response (2, 31). Several studies have demonstrated that the stimulation of NMDA receptors (such as glutamate) induce an anxiogenic-like behavior in a variety of animal models of anxiety (32, 33). Competitive and non-competitive NMDA receptor antagonists induce anxiolytic behaviors in human and laboratory animals (34). The anxiolytic effects of nZnO and cZnO may work through inhibition of NMDA receptors.

An alternative mechanism is related to the gamma- aminobutyric acid (GABA) neurotransmitter. Zn++ promotes the release of GABA from interneurons in the hippocampus, thus enhancing the inhibitory effects of this neurotransmitter and leading to a decrease in the pre-synaptic release of glutamate (35). Thus release of Zn++ from cZnO and nZnO may reduce anxiety via reduced glutamate release and inactivation of NMDA receptors (31, 35).

The opioid receptor agonists and antagonists, such as morphine and naloxone, tend to induce anxiolytic and anxiogenic responses respectively (17, 36-39); findings supported by our results when morphine and naloxone were injected peripherally. In our study naloxone (1 mg/kg, IP) did not inhibit the anxiolytic effects of cZnO and nZnO and morphine (6 mg/kg, IP) increased the anxiolytic effect of nZnO and cZnO. It is possible that shared or unshared pathways have been used by morphine and ZnO to induce this higher anxiolytic effect.

According to a previous study there is an interaction between morphine and the glutamatergic system (40). It has been shown that an acute injection of morphine decreased the level of extracellular glutamate in the brain (40). Electrophysiological studies have shown that there is a relationship between NMDA receptor subunits and mu-opioid receptors in the CNS (41). NMDA receptor antagonists disrupt the development of morphine tolerance (42) and demonstrate an anxiolytic effect (17). Zinc also modulates the activity of this receptor and reduces glutamate activity via the pathways mentioned previously (19, 31, 35).

The GABAergic system is a possible common pathway for the additive anxiolytic effect of morphine and ZnO. Various studies have indicated that the opioidergic system interacts to modulate anxiety-related behavior through the GABAergic system in some specific brain areas (43, 44) and there is an interaction between intra cellular zinc and GABAergic system activity (35).

Our data show that the increased anxiolytic effect of morphine and nZnO is higher than that of cZnO. This may be related to the small size of the nanoparticles that facilitate the distribution of ZnO particles to different regions (26, 28).

The intra CA1 injection of naloxone (1 μg/rat) alone did not affect the anxiety indexes in our investigation, although previous studies have shown that this dose of naloxone completely blocked opioid receptors in the hippocampus (25). In the presence of naloxone (1 μg/rat), the anxiolytic effect of nZnO was maintained but cZnO was prevented from inducing its anxiolytic effect. Probably this is due to the physiochemical properties of the nano particles (45).

## Conclusion

Our results suggest that zinc oxide supplements may be effective for the reduction of anxiety and that opioidergic system activity can influence their anxiolytic effects through shared or unshared mechanisms. It is possible that other neurochemical systems are involved in this phenomenon. This area of research requires further investigation.
